# Human Cancer Long Non-Coding RNA Transcriptomes

**DOI:** 10.1371/journal.pone.0025915

**Published:** 2011-10-03

**Authors:** Ewan A. Gibb, Emily A. Vucic, Katey S. S. Enfield, Greg L. Stewart, Kim M. Lonergan, Jennifer Y. Kennett, Daiana D. Becker-Santos, Calum E. MacAulay, Stephen Lam, Carolyn J. Brown, Wan L. Lam

**Affiliations:** 1 British Columbia Cancer Agency Research Centre, Vancouver, British Columbia, Canada; 2 Department of Pathology and Laboratory Medicine, University of British Columbia, Vancouver, British Columbia, Canada; 3 Interdisciplinary Oncology Program, University of British Columbia, Vancouver, British Columbia, Canada; 4 Department of Medical Genetics, University of British Columbia, Vancouver, British Columbia, Canada; National Cancer Institute, United States of America

## Abstract

Once thought to be a part of the ‘dark matter’ of the genome, long non-coding RNAs (lncRNAs) are emerging as an integral functional component of the mammalian transcriptome. LncRNAs are a novel class of mRNA-like transcripts which, despite no known protein-coding potential, demonstrate a wide range of structural and functional roles in cellular biology. However, the magnitude of the contribution of lncRNA expression to normal human tissues and cancers has not been investigated in a comprehensive manner. In this study, we compiled 272 human serial analysis of gene expression (SAGE) libraries to delineate lncRNA transcription patterns across a broad spectrum of normal human tissues and cancers. Using a novel lncRNA discovery pipeline we parsed over 24 million SAGE tags and report lncRNA expression profiles across a panel of 26 different normal human tissues and 19 human cancers. Our findings show extensive, tissue-specific lncRNA expression in normal tissues and highly aberrant lncRNA expression in human cancers. Here, we present a first generation atlas for lncRNA profiling in cancer.

## Introduction

Genome instability and mutation are a hallmark of cancer [1]. Genetic and epigenetic changes result in aberrant expression of protein-coding genes and many classes of non-coding RNAs (ncRNAs), including microRNAs (miRNAs). MiRNAs have proven to be major players in human carcinogenesis, despite comprising only a small fraction of ncRNAs [2].

Once thought to be the ‘dark matter’ of the genome, ncRNAs have emerged as an integral component of the mammalian transcriptome [3], [4], [5]. These enigmatic molecules are defined by lack of protein-coding sequence, yet can play both structural and functional roles in the cell [6], [7]. NcRNAs can been grouped into two major classes, the small ncRNAs, which include miRNAs and other non-coding transcripts of less than 200 nucleotides (nt), and the more recently described lncRNAs, which range from 200 nt to >100 kilobases (kb) [8].

LncRNAs can be intergenic, intronic, antisense or overlapping with protein-coding genes or other ncRNAs [9], [10], [11], [12]. The known repertoire of lncRNA functions is rapidly expanding – with demonstrated roles as mediators of mRNA decay [13], structural scaffolds for nuclear substructures [14], [15], as host genes for miRNAs [16], [17], and as regulators of chromatin remodeling [18], [19], [20], [21] – even though the functional identities of many lncRNAs have yet to be uncovered [6], [7], [22]. Recently, human cancers have been described to have altered expression of satellite repeats [23], transcribed ultra conserved regions (T-UCRs) [24], and antisense transcripts [25]. Beyond expression changes, accumulating evidence indicates aberrant expression of lncRNAs may play an important functional role in cancer biology [26], [27], [28]. The well-studied HOX antisense intergenic RNA (*HOTAIR*), for example, is highly expressed in breast cancers and breast cancer metastases and plays a role in retargeting chromatin remodeling complexes [29]. Similarly, high expression of the nuclear speckle associated lncRNA metastasis-associated lung adenocarcinoma transcript 1 (*MALAT1*) modulates alternative splicing and has been associated with metastasis and poor outcome in patients with lung cancer [30], [31]. While these examples are intriguing, the extent of the contribution of differential lncRNA expression to human cancer is currently unknown.

With a conservative estimate of 23,000 lncRNAs in the human genome, these transcripts rival the ∼20,000 protein-coding genes [5], [11], [32], [33]. Over the past two decades, microarray profiling has generated a wealth of information on protein-coding gene expression patterns in human cancers. However, as lncRNA specific probes are underrepresented on commercial microarrays used in cancer transcriptome profiling, these data do not apply to ncRNAs. Global sequencing of RNA populations is a new approach used to profile RNA expression levels that will capture the extent of lncRNA expression. Recently, genome-wide ncRNA expression profiles were determined in 11 samples representing different types of human tissues [34].

One sequence-based method for enumerating the abundance of polyadenylated transcripts is SAGE [35]. As many lncRNAs themselves are polyadenylated, lncRNA transcript levels can be deduced by way of direct enumeration of corresponding sequence tags using SAGE technology. In fact, two antisense lncRNAs were discovered using a SAGE-based method [25]. Since the invention of SAGE technology in the mid 1990s, numerous SAGE libraries representing a diversity of human and mouse, normal and malignant tissues and cell lines have become publically available [36]. Of the 755 human SAGE libraries in the Gene Expression Omnibus (GEO) database, ∼276 include SAGE libraries derived from human cancers or dysplasias [37].

In this study, we compiled 272 human SAGE libraries to delineate lncRNA transcription patterns across a broad spectrum of human tissues and cancers. Using a custom lncRNA discovery pipeline, we parsed over 24 million SAGE sequence tags to deduce (1) the specific lncRNA expression patterns in 26 human tissues and discovered ubiquitously expressed as well as tissue specific lncRNAs, and (2) the aberrant expression patterns of lncRNAs in 19 human cancers.

## Results

### Assembling human SAGE libraries of normal and cancer tissues

**Figure 1:**
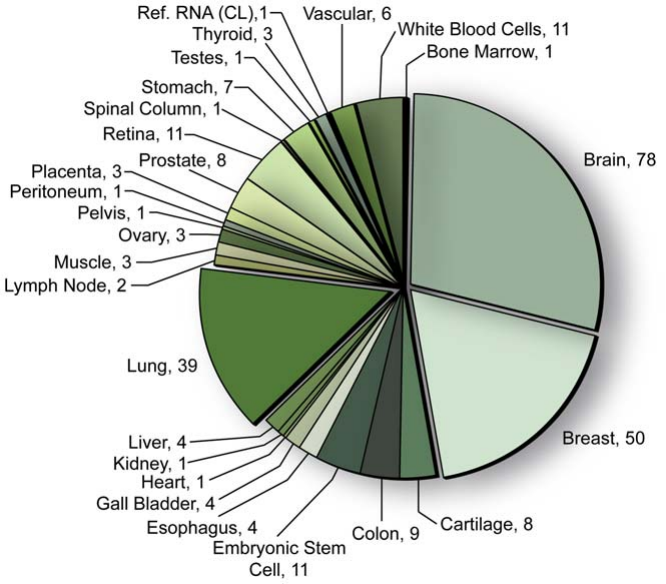
Tissue-type distribution of the 272 SAGE libraries with a minimum raw tag count of 50,000. (CL) indicates one SAGE library that was generated from a mixture of human cell lines.

A total of 1,824 SAGE libraries (in short SAGE, long SAGE and SAGE-seq format) of human and non-human origins are publically available via GEO. To explore lncRNA expression in the broadest range of human tissue types and cancer types, we downloaded 360 GEO accessioned human short SAGE libraries comprised of libraries curated by the Cancer Genome Anatomy Project (324 libraries) and lung tissue and cancer datasets (36 libraries) (Table S1). Individual libraries were filtered for sequence depth, retaining only those libraries with >50,000 raw tags, to provide 272 SAGE libraries for analysis using our lncRNA discovery pipeline (Table S2). The 272 SAGE libraries are comprised of a total of 24,436,076 raw sequence tags with an average raw tag count of 90,212 per library. Collectively, the libraries spanned 26 normal human tissue types, including 19 human cancer types, and 9 tissue types derived from cell line libraries (Figure 1, Table S3).

### Long non-coding RNA discovery pipeline

**Figure 2:**
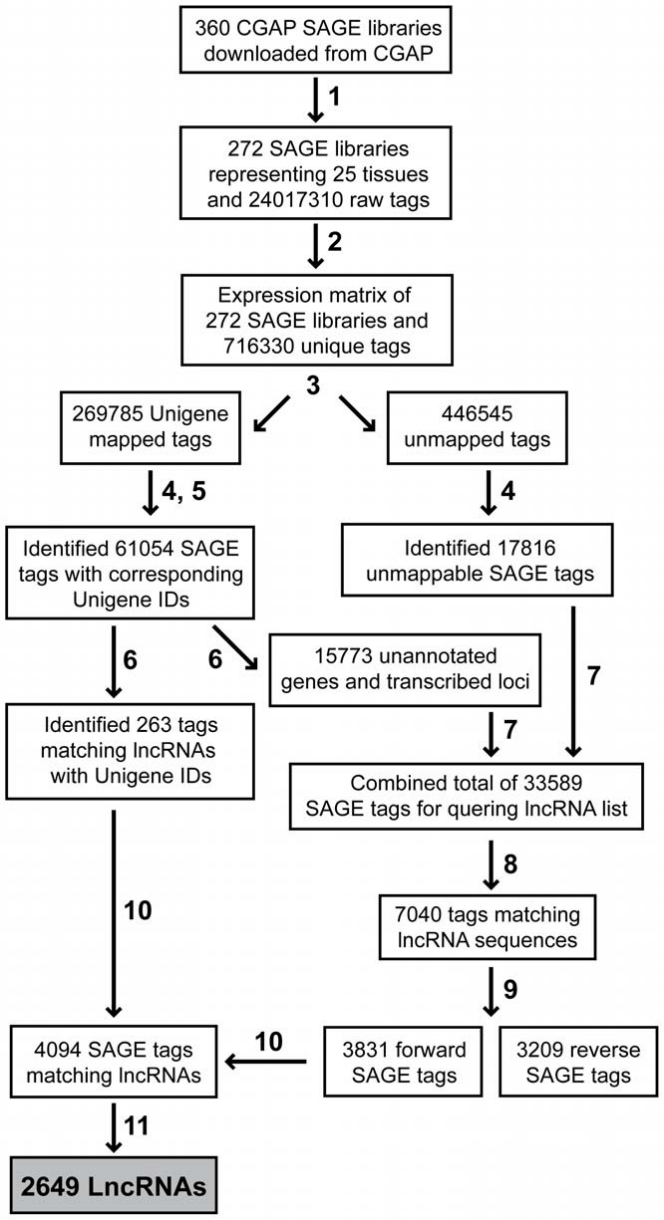
LncRNA discovery pipeline using SAGE analysis. Numbers indicate programs or filtering steps as follows: (1) filtering to retain only those libraries with a minimum of 50,000 raw tag counts, (2) identifying unique SAGE tags and constructing SAGE tag expression matrix, (3) mapping SAGE tags to Unigene IDs using SAGE Genie mapping files, (4) filtering lists to retain only tags with ≥2 raw counts in a ≥3 of 272 libraries, (5) determining gene identity using SAGE Genie, (6) separating Unigene tags mapping to lncRNAs and ambiguous transcripts, (7) pooling ambiguous tags and unmapped tags, (8) mapping sequence tags to the reference list of 9,891 lncRNAs using SeqMap, a tag-to-gene mapping program, (remaining tags may map to unannotated lncRNAs or antisense transcripts not included in our reference list) (9) filtering tag matches for strand sense, (10) pooling forward mapping tags and tags determined from Unigene, and (11) confirming tag-to-lncRNA matches and summing tag counts for lncRNAs with multiple tag matches. A complete list of lncRNAs is provided as Table S5 and tag-to-lncRNA matches are provided as Table S6.

To generate lncRNA expression profiles, we developed a lncRNA discovery pipeline to map tag-to-lncRNA matches (Figure 2). A SAGE tag expression matrix was constructed from all unique tags (n = 716,330) identified within the dataset of 272 libraries. Unigene mapped and unmapped SAGE tags (n = 269,785 and n = 446,545, respectively) were separated into distinct expression matrices which were subsequently filtered to retain only those tags with at least 2 raw tag counts in 3 or more SAGE libraries. Using SAGE Genie to assign gene identifiers to the Unigene IDs, 263 of the 61,054 filtered tags with corresponding Unigene IDs mapped to known lncRNAs, and 15,773 tags either lacked gene names or had ambiguous annotations (e.g. transcribed loci, cDNAs, hypothetical genes). Based on the absence of confirmed association with known genes, these 15,773 tag-to-Unigene ID matches were considered as candidate lncRNA tags.

The 15,773 Unigene tags with ambiguous gene identifiers were combined with the 17,816 unmapped, filtered tags for a total of 33,589 SAGE tags with the potential to generate tag-to-lncRNA matches. Using SeqMap, we mapped 7,040 of the 33,589 tags to lncRNA sequences from the reference lncRNA list (Table S4). The proportion of tag-to-lncRNA matches is consistent with the fact that our reference list of 9,891 lncRNAs represents only a portion of the estimated 23,000 lncRNAs in the genome [33]. The remaining tags that do not map to lncRNAs from our reference list may represent antisense transcripts to protein-coding genes or other ncRNAs which were filtered.

Of the 7,040 lncRNA tag matches, 3,831 mapped in the forward orientation, while 3,209 mapped in the reverse direction. In SAGE, tags matching transcript in the forward orientation are likely derived from that transcript, while tags matching in the reverse orientation are not. This is true regardless of whether the gene is normally transcribed from the plus or minus DNA strand. In this study, we were interested in the expression profiles of a curated set of lncRNAs, rather than novel gene discovery. As reverse tag matches do not corroborate the expression of the lncRNAs described herein, these tags were excluded from further analysis.

The 3,831 tags newly mapped to lncRNAs were combined with the 263 tags identified from Unigene mapping for a total of 4,094 tags uniquely mapping to lncRNAs. Where multiple tags mapped to a distinct lncRNA, the tags were collapsed by summing the tag counts to capture all transcript variants and isoforms. The end result was a lncRNA expression matrix consisting of 2,649 distinct lncRNAs (Tables S5 and S6). The lncRNAs with the highest expression were detectable in the majority (>90%) of the 272 libraries (Table 1). These included characterized examples such as nuclear paraspeckle assembly transcript 1 (*NEAT1*) and growth arrest-specific 5 (*GAS5*).

**Table 1:** The eleven most highly expressed lncRNAs detected in >90% of the 272 SAGE libraries.

Gene Name	Ensembl Gene	Chr	Start (bp)	End (bp)	Strand
*MALAT1*	ENSG00000251562	11	65265233	65273940	1
*GAS5*	ENSG00000234741	1	173833038	173838020	−1
*NEAT1*	ENSG00000245532	11	65190245	65213011	1
NCRNA00188	ENSG00000175061	17	16342289	16367300	1
RP11-425M5.7	ENSG00000225759	20	36247700	36251521	−1
*SNHG6*	ENSG00000245910	8	67833919	67838633	−1
*SNHG5*	ENSG00000203875	6	86386725	86388451	−1
*SCAND2*	ENSG00000176700	15	85174682	85185695	1
AC104759.1	ENSG00000246638	15	31685046	31696932	1
AC002472.9	ENSG00000230513	22	21356175	21364631	1
AC090937.2	ENSG00000225733	3	14961854	14989931	−1

Also see Table S5.

### Long non-coding RNA expression profiles in normal human tissues

**Figure 3:**
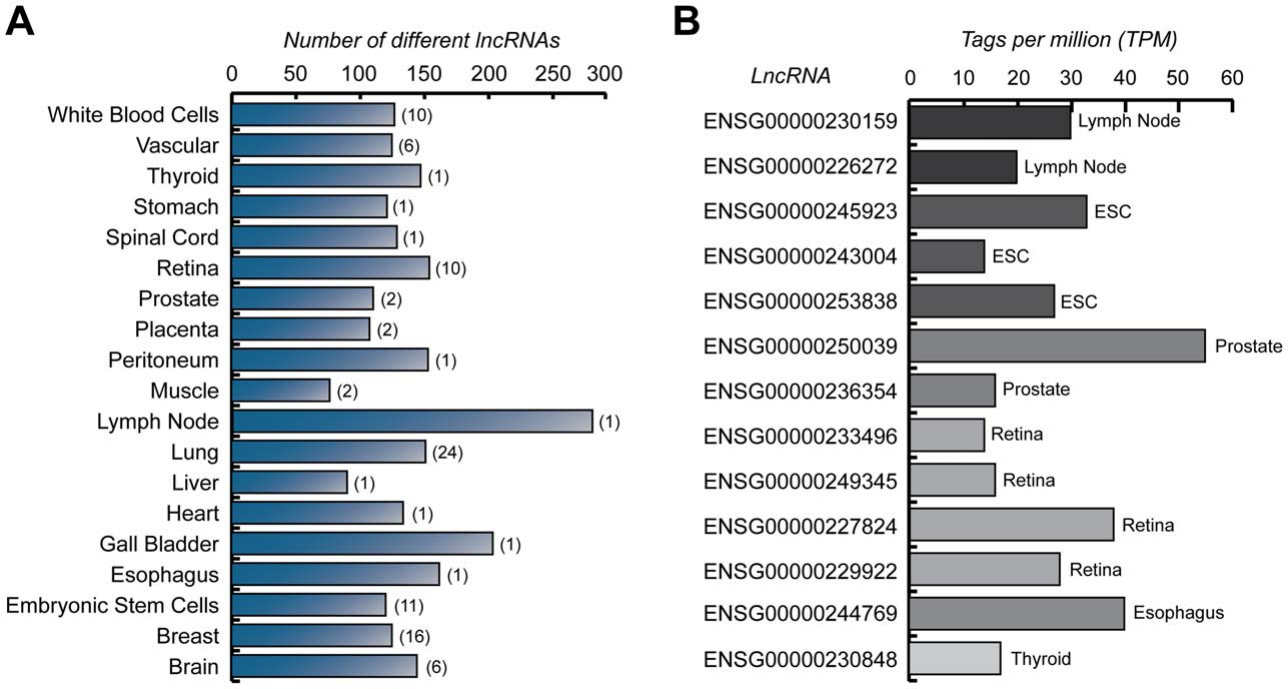
Distribution and levels of lncRNA expression in normal human tissues. (A) Number of distinct lncRNAs expressed in normal human tissues, white blood cells and embryonic stem cells with a minimum average TPM of 20. The values in brackets indicate the number of SAGE libraries for each tissue. (B) Examples of lncRNAs detected exclusively in a single normal human tissue or in embryonic stem cells (ESC) with a minimum expression level of 10 TPM. For tissues with two or more libraries, the TPM values were averaged. LncRNAs without names are labeled with an Ensembl ID.

Of the 272 SAGE libraries, 72 represented normal human tissues. Expression of lncRNAs was detected in all tissue types, although the number of unique lncRNAs detected varied considerably (Figure 3A). On average, there were 145 distinct lncRNAs with a mean tags per million (TPM) of 20 detected in each tissue. Tissues such as lymph node and gall bladder showed the highest number of distinct lncRNAs, while the lowest numbers of distinct lncRNAs were found in the muscle and liver.

**Figure 4:**
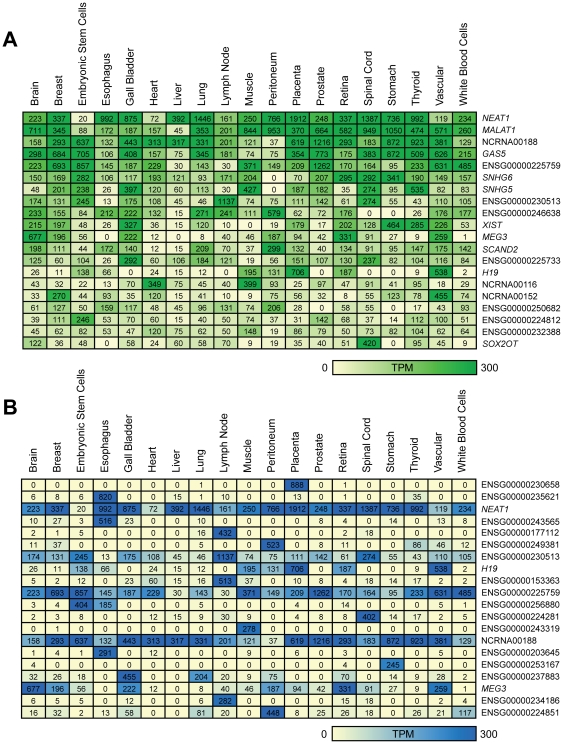
Expression patterns of lncRNAs in normal human tissues. (A) LncRNAs with the highest overall expression (B) LncRNAs with the highest variance by a coefficient of variation (CV) test. Heatmaps indicate the relative intensity (normalized TPM) of each lncRNA across seventeen human tissues, white blood cells and human embryonic stem cells. Where more than one SAGE library was available, the TPM values were averaged. For the heatmap, the maximum threshold was set at 300 TPM. LncRNAs without names are labeled with an Ensembl ID.

We next focused on these libraries to determine whether tissue-specific lncRNA expression profiles could be generated (Table S7). Figure 4A shows the top 20 most highly expressed lncRNAs detected in the panel of normal tissues. Distinct lncRNAs detected at high expression levels in normal tissues included those characterized in the literature such as *NEAT1*, *GAS5* and X-inactive-specific transcript (*XIST*). However, at least half of the highly expressed lncRNAs are novel and currently uncharacterized. To confirm the lncRNA expression profiles, we queried the expression patterns of the most highly expressed lncRNAs using RNASeq data from the Illumina Human BodyMap 2.0 project. This data was recently added to Ensembl release 62 and is presented as an optional track. Of our most highly expressed lncRNAs, the majority were widely expressed in the tissue samples from the Illumina dataset, consistent with our findings (Table S8, Figures S1 and S2). Concurrently, lncRNA expression was also found to be highly variable, with each human tissue having a unique lncRNA expression pattern (Figure 4B). Intriguingly, a number of lncRNAs were expressed in a tissue-exclusive manner (Figure 3B).

### Long non-coding RNA expression profiles in human cancers

**Figure 5:**
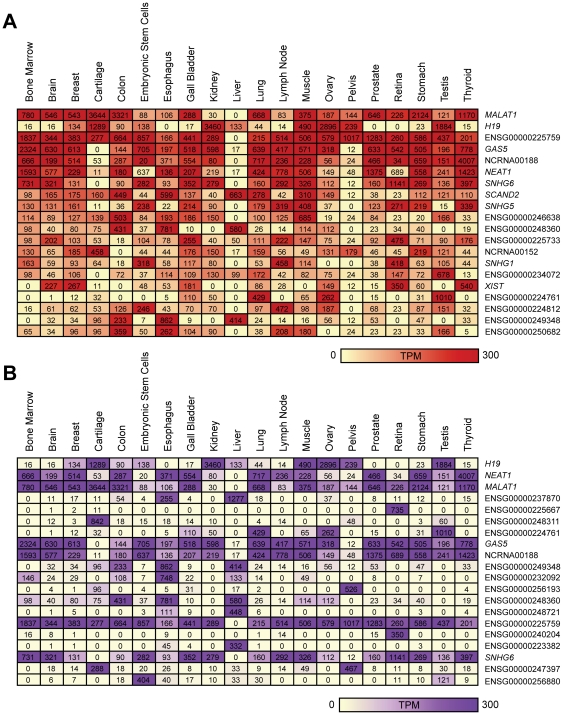
Expression patterns of lncRNAs in human cancers. (A) LncRNAs with the highest overall expression (B) LncRNAs with the highest variance by a coefficient of variation (CV) test. Heatmaps indicate the relative intensity (normalized TPM) of each lncRNA across seventeen human cancers and human embryonic stem cells. Where more than one SAGE library was available, the TPM values were averaged. For the heatmap, the maximum threshold was set at 300 TPM. LncRNAs without names are labeled with an Ensembl ID.

Aberrant protein-coding gene expression is well described in cancer. However, aberrant expression of ncRNAs, including miRNAs and lncRNAs, has only recently been associated with this disease [2], [26], [27], [38]. To delineate lncRNA expression profiles associated with human cancers, we created a human cancer expression matrix based on 167 cancer SAGE libraries included in our dataset (Table S9). For the lung cancer dataset, metaplasia, dysplasia and inflammatory tissues were excluded from analysis as these represent precancerous stages [39], [40]. Figure 5A shows the top 20 most highly expressed lncRNAs across the profiled cancers. Like the normal tissues, lncRNA expression in human cancer was also found to be highly variable (Figure 5B).

### Human cancers demonstrate significantly altered lncRNA expression patterns

**Figure 6:**
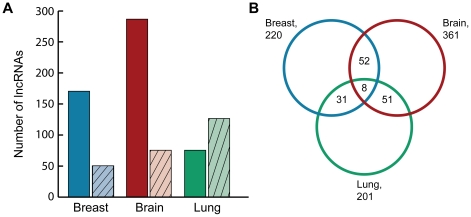
Aberrantly expressed lncRNAs in human cancers. (A) Number of lncRNAs showing significant expression changes. The number of lncRNAs determined to have significant (BH p-value <0.05) differential expression of 2-fold or greater reported. Solid bars indicate upregulated genes, while bars with hatch marks indicate downregulated genes (B) Venn diagram of differentially expressed lncRNAs in human carcinomas.

To determine the extent of differential lncRNA expression in human cancer, we created three expression matrices for each breast, brain and lung cancer which included a minimum of five normal and five cancer SAGE libraries (Table S10). The breast, brain and lung lncRNA expression matrices were independently sorted for significant and differentially expressed lncRNAs (p-value<0.05, ≥2-fold expression change based on a non-parametric permutation test [41]). In each type of cancer, we found at least 200 lncRNAs to have significant differential expression based on these criteria (Figure 6A). Intriguingly, there was overlap between the lncRNAs that were differentially expressed in each tissue (Figure 6B), including 8 lncRNAs that were differentially expressed in all three cancers (Table 2). The ten most up- and down-regulated lncRNAs for each cancer are found in Table S11.

**Table 2:** Aberrantly expressed lncRNAs common to brain, breast and lung cancers.

						Fold Change			Corrected p-value		
lncRNA	Ensembl Gene ID	Chr	Start	End	Strand	Brain	Breast	Lung	Brain	Breast	Lung
AC058791.1	ENSG00000230937	7	130565751	130598069	−1	7.00	−3.00	3.59	0.00122	0.02373	0.00000
CTA-55I10.1	ENSG00000255717	1	209602165	209606183	1	3.37	−2.05	2.72	0.00041	0.00190	0.00000
NCRNA00263	ENSG00000247556	10	102133372	102143125	1	12.37	2.10	2.46	0.00004	0.00056	0.00000
AC080037.2	ENSG00000245411	17	70594180	70636611	−1	3.08	2.76	−2.14	0.00009	0.00027	0.00141
AC012652.1	ENSG00000226380	15	41576203	41601901	1	6.45	3.53	−2.33	0.00026	0.00018	0.03657
RP11-18C24.6	ENSG00000253288	12	120928131	120933743	−1	−2.48	−3.13	−2.86	0.00405	0.00639	0.01311
RP11-238K6.1	ENSG00000235823	8	138821687	139095813	−1	7.07	4.18	−4.35	0.00529	0.00012	0.00037
SNHG1	ENSG00000248008	11	62619460	62623386	−1	3.04	3.27	−5.03	0.00403	0.00043	0.00003

### Chromosomal distribution of long non-coding RNAs

**Figure 7:**
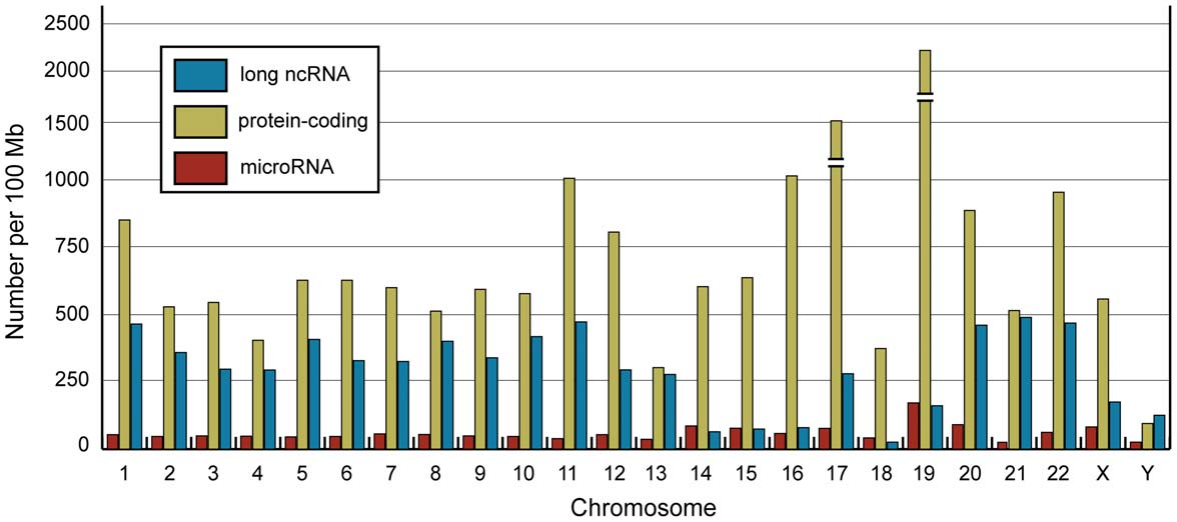
Chromosomal distribution of protein-coding genes, microRNAs and long non-coding RNAs in the human genome. Protein-coding gene (n = 20,655), microRNA (n = 1,746) and long non-coding RNA (n = 9,891) coordinates were downloaded from Ensembl v62 using BioMart.

We constructed a distribution plot to determine the chromosomal distribution of the 9,891 lncRNA genes in our lncRNA reference list (Table S3). The lncRNAs are distributed throughout the genome and are present on every chromosome (Figure 7). Protein-coding genes and miRNAs appear to share a similar chromosome distribution (Spearman correlation p>0.05, Figure S3A). However, the chromosome distribution of lncRNAs did not correlate with either protein-coding genes or miRNAs (Spearman correlation p<0.05, Figures S3B, S3C).

## Discussion

In recent years, the concept of the functional genome has been re-written to include a multitude of newly discovered classes of ncRNA transcripts [42], [43], [44], [45]. Although the functional significance of long non-coding RNAs has long been recognized [46], [47], the abundance and scale of lncRNA expression changes in cancer is just beginning to come to light. For this reason, charting the transcriptional landscape of lncRNAs across human tissue and cancer types is a key step in understanding lncRNA functional significance in cancer.

Here, we present the first multi-tissue, cross-cancer lncRNA expression profiling study. Large-scale expression profiling datasets, such as SAGE, represent a valuable resource for investigating the expression pattern of polyadenylated lncRNAs. While this approach excludes the profiling of non-polyadenylated lncRNAs, it nonetheless facilitates the simultaneous profiling of thousands of polyadenylated lncRNAs in a wide range of human tissues and cancers. Using 272 SAGE libraries, representing 26 non-malignant human tissues, 19 human cancer types and 9 cancer cell lines, we have produced a first generation atlas of cross-cancer lncRNA expression profiles as a resource for this fast growing area of cancer research. Current estimates of the number of lncRNAs encoded in the human genome vary widely, ranging from ∼7,000 to 23,000 or more [7]. These estimates rival the abundance of the estimated 20,000+ protein-coding genes. Our analysis showed that lncRNAs are distributed on all 22 autosomes and sex chromosomes, yet the distribution pattern did not correlate with either protein-coding genes or miRNAs (Figure 7, Figure S3).

Examination of 72 SAGE libraries of normal human tissues revealed lncRNA expression in brain, breast, esophagus, gall bladder, heart, liver, lung, lymph node, muscle, peritoneum, placenta, prostate, retina, spinal cord, stomach, thyroid, vascular tissue, embryonic stem cells and white blood cells. We find extensive and highly differential patterns of lncRNA expression in normal human tissues (Figures 3 and 4), corroborating a previous report of tissue-specific ncRNA patterns [34]. For example, the lncRNA NCRNA00116 was highly expressed in the contractile tissues, namely heart (TPM = 349) and muscle (TPM = 399). LncRNAs ENSG00000230658 and ENSG00000235621 showed very high expression (TPM = 888) in placenta and esophagus (TPM = 820) respectively, but low or undetectable expression in other tissues, which may indicate a tissue-specific role for these transcripts. The brain-associated and putative tumor suppressor lncRNA maternally expressed 3 (*MEG3*) [48], displayed the highest expression in brain in our dataset (TPM = 677), but showed low level expression in other tissue types (Figure 4). Collectively, these data suggest some lncRNAs may function in a tissue-specific manner.

Only ∼1% of the lncRNAs were ubiquitously expressed across all tissues examined. These constantly expressed lncRNAs are reminiscent of the expression patterns of “housekeeping” protein-coding genes [49]. The eleven lncRNAs in Table 1 were expressed in at least 90% of 272 SAGE libraries in our dataset, implicating that these transcripts may participate in common biological processes. However, the absolute expression level varied for each tissue, sometimes by hundreds of TPM (Figure 4). This suggests certain lncRNAs may be required at different cellular levels in different tissues or under different conditions, much like many constitutively expressed protein-coding genes [50], [51], [52]. The concept of lncRNAs functioning as constitutively expressed regulators has been previously proposed. For example, the lncRNA *XIST* is critical for female development due to its functional role in X-chromosome inactivation [47], [53]. Concordantly, a number of the most highly and frequently expressed lncRNAs in our dataset have prior associations with key biological processes, including *NEAT1*, a structural scaffold for paraspeckle formation [14], [54], *MALAT1* which regulates alternative splicing [31] and small nucleolar RNA host gene 6 (*SNHG6*) which hosts a snoRNA, which function in RNA modification [55]. These findings suggest that lncRNAs may be critical to normal tissue maintenance and function.

In this cross-cancer type analysis, we found that lncRNAs aberrantly expressed in a specific cancer may also be altered in other cancers. For example, while *MEG3* is highly expressed in normal brain tissues, this lncRNA was strongly decreased in our brain cancer datasets, and strikingly so in gall bladder, retinal and prostate cancers, consistent with the proposed tumor suppressor role for *MEG3* [48], [56], [57]. In another example, miR155 host gene (*miR155HG*), a lncRNA processed to the miRNA *miR-155*, was highly overexpressed in B-cell lymphoma consistent with previous reports [16], but also was also upregulated in esophageal and gall bladder cancers.

Long non-coding RNAs are also implicated in the regulation of embryogenesis [58], [59], [60]. Fetal lncRNAs reactivated in cancers may represent critical regulators of pluripotency or cellular growth. For example, the lncRNA urothelial cancer associated 1 (*UCA1*) has demonstrated roles in both embryonic development and is implicated in bladder cancer, supporting this concept [61]. In our datasets, we found several lncRNAs with low expression in normal tissues, but with high expression in both embryonic stem cells and cancer (Table S12). While these reactivated fetal lncRNAs represented mostly uncharacterized examples, *H19*, a well-studied lncRNA with associations in both mammalian development and cancer [53], was also detected in our dataset. Interestingly, *NEAT1*, which is constitutively and highly expressed in normal tissues [34], [62], with the exception of embryonic stem cells, was downregulated in lung, liver, esophageal and retinal cancers (retinoblastoma).

Since genomic amplifications and deletions are key mechanisms of gene deregulation in cancer, we investigated changes in lncRNA expression in genomic regions frequently altered in breast, brain and lung cancer. Comparison of the significantly (p<0.05) deregulated lncRNAs common between brain, breast and lung cancer tissues revealed eight lncRNAs were differentially regulated (≥2-fold) compared to normal tissue. Intriguingly, three of these lncRNAs - ENSG00000226380, ENSG00000230937 and ENSG00000253288 - were located on 7q32.3, 1q32.2, and 8q24.23, respectively, in regions completely devoid of protein-coding genes. Like protein-coding genes and miRNAs, it is possible that differential lncRNA expression is driven by similar mechanisms of disruption, including copy number gain/loss or aberrant methylation patterns. Indeed, high level amplification of lncRNA containing loci such as cytoband 19p12 has been reported in breast cancer [63], while high level amplification of 12p13.2 (which contains a number of lncRNA loci) has been reported in breast cancer, glioblastoma, astrocytoma, and squamous cell lung cancer [64], [65], [66], [67]. Likewise, aberrant expression of a number of lncRNAs has been tied to altered methylation patterns [68], [69]. However, the mechanism(s) driving aberrant lncRNA expression remains mostly unknown.

While lncRNAs have been documented for nearly three decades, the magnitude and diversity of lncRNA expression has only recently been appreciated. It is estimated that lncRNAs in the human genome number into the tens of thousands, effectively doubling the number of potential gene targets in cancer gene expression networks. Large scale, cross-tissue and cancer studies are crucial to understanding the regulation of lncRNA expression and how these novel transcripts integrate with our current understanding of the mammalian transcriptome. Moreover, a deeper understanding of lncRNA expression will not only expand the number of potential target cancer genes, but also facilitate development of novel anti-cancer therapies, such as gene regulation mediated by antisense RNAs [70] or targeting lncRNA-protein interactions [28].

## Materials and Methods

### SAGE Libraries

This study uses publically available SAGE libraries for data analysis. A total of 360 SAGE libraries, including 324 from the Cancer Genome Anatomy Project (CGAP) SAGE library collection (GSE15309), 19 lung bronchial epithelium libraries (GSE3707), 13 lung cancer libraries (GSE7898) and 4 never smoker bronchial epithelium libraries (GSE5473), were downloaded from GEO (Table S1). Libraries constructed from non-human samples, as well as long SAGE and SAGE-seq libraries were not used in this study. To facilitate direct comparison the SAGE libraries were filtered to retain only those libraries with >50,000 raw tag counts resulting in 272 libraries suitable for analysis (Table S2).

### Long non-coding RNA reference list

The lncRNA discovery pipeline is based on a reference list of human lncRNAs curated by the online genomic database Ensembl release 62, built on the Genome Reference Consortium release GRCh37 [71]. The lncRNA reference list was compiled from 1,239 Ensembl (v62) IDs designated as ‘lincRNAs’ (long intergenic non-coding RNAs, a subclass of lncRNAs) and 8,652 Ensembl IDs (v62) designated as ‘processed transcripts’ for a total of 9,891 lncRNAs (Table S4). All the lncRNAs used to query the SAGE libraries were Ensembl curated transcripts without a predicted open reading frame. The sequences of all lncRNA transcripts were retrieved from Ensembl (v62) using the Biomart data management system.

### SAGE tag-to-gene mapping

Custom Perl scripts were used to create an expression matrix of the unique SAGE tags across the 272 libraries (Perl scripts: getuniquetags.pl and makeTable_April20.pl). The SAGE tags were mapped to Unigene IDs using custom Perl scripts and a short SAGE mapping file (mapping file: Hs_short) downloaded from SAGE Genie (http://cgap.nci.nih.gov/SAGE), to create a matrix of Unigene ID mapped tags and a matrix of unmapped tags (Perl script: extractUnmappedTags_Unigene). The two expression matrices of unmapped tags and Unigene mapped tags were independently filtered to retain only tags with raw tag counts of 2 or more, appearing in at least 3 SAGE libraries.

For the Unigene mapped tags, gene identifiers were assigned to Unigene IDs using SAGE Genie. From this dataset, tags matching known or candidate lncRNAs were extracted manually. Candidate lncRNAs are Unigene IDs with no gene name or matching one or more of the following descriptors: ‘non-coding’, ‘non-protein’, ‘cDNA’, ‘transcribed locus’, ‘clone IMAGE’, ‘chr(#)orf(#)’, ‘hypothetical’, ‘family with sequence similarity’, ‘FLJ(#)’, or ‘KIAA(#)’. The candidate lncRNA tags were merged with the unmapped tags and used as a single dataset from which to identify sequence matches to the lncRNA reference list.

The tag-to-gene mapping program SeqMap was used to identify perfect (0 mismatches) tag matches to the transcript sequences from the reference lncRNA list. Tags mapping to lncRNAs were filtered to retain those corresponding to the forward (‘sense’) strand, while reverse tag matches do not corroborate the expression of the candidate lncRNAs and were not analyzed further. The forward strand tags that mapped to lncRNAs were then combined with the Unigene tags that mapped to lncRNAs to create an expression matrix of SAGE tags mapping to lncRNAs. This matrix was remapped to the lncRNA reference list to confirm accurate tag-to-lncRNA matches.

### Data pre-processing

In cases where multiple tags mapped to the same lncRNA, the tags were compressed by summing the tag counts to capture all lncRNA transcript variants and isoforms (Perl script: sumRows.pl). SAGE tags mapping to more than one lncRNA were discarded. Raw tag counts for each SAGE library were normalized to TPM to facilitate adequate comparison among libraries. Additional expression matrices included only SAGE libraries of interest for a given analysis, while removing any columns with unwanted SAGE libraries. These submatrices were filtered to remove lncRNAs with undetected expression. When a tissue or cancer was represented by more than one SAGE library, the normalized TPM were averaged. Finally, all Ensembl v62 IDs were lifted to Ensembl v63, any missing or reassigned IDs were removed from the final lncRNA list.

### Statistical analysis

To ensure statistical significance when comparing normal tissues with cancerous tissues, the lncRNA expression matrix was filtered to retain only those tissues represented by a minimum of 5 normal and 5 cancer SAGE libraries. These SAGE libraries were used to derive cancer specific expression matrices. To compare the expression of lncRNAs between normal libraries and cancer libraries, we performed a normalization of expression by permutation of SAGE (*NEPS*) test as described [41]. LncRNAs with permutation scores of >0.05 were considered to be statistically significant. All fold changes were calculated by dividing the average expression of the cancer SAGE libraries by the average expression of the normal SAGE libraries. Variance calculations were performed by calculating the coefficient of variation (CV) across the averaged normal or cancer SAGE libraries. The lncRNA distribution plots were created by normalizing the number of lncRNAs, miRNAs, or protein-coding genes to 100 megabase (MB) of chromosome and then performing a Spearman correlation.

## Supporting Information

Figure S1 Tissue expression profiles of *MALAT1*. Expression was derived from the Human BodyMap 2.0 RNASeq track in Ensembl v62.
(JPG)

Figure S2 Tissue expression profiles of NCRNA00188. Expression was derived from the Human BodyMap 2.0 RNASeq track in Ensembl v62.
(JPG)

Figure S3 Correlation of chromosome distribution between protein-coding genes, miRNAs and lncRNAs. (A) Protein-coding genes compared to miRNAs, (B) Protein-coding genes compared to lncRNAs, (C) lncRNAs compared to miRNAs. The chromosome locations of protein-coding genes (n = 20,655), microRNAs (n = 1746) and long non-coding RNAs (n = 9,891) were downloaded from Ensembl v62. The graphs were generated using GraphPad Prism.
(JPG)

Table S1 GEO Libraries.
(XLSX)

Table S2 Filtered SAGE libraries.
(XLSX)

Table S3 SAGE library information.
(DOC)

Table S4 LncRNA reference list.
(XLSX)

Table S5 LncRNA expression matrix.
(XLSX)

Table S6 Tag-to-lncRNA matches.
(XLSX)

Table S7 Normal tissue lncRNA expression matrix.
(XLSX)

Table S8 Expression validation by BodyMap RNASeq.
(XLSX)

Table S9 Cancer tissue lncRNA expression matrix.
(XLSX)

Table S10 Brain, breast and lung libraries.
(XLS)

Table S11 Top differentially expressed brain, breast and lung lncRNAs.
(XLSX)

Table S12 ESC and cancers.
(XLSX)
