# Supplementary figures and images for: Human Cancer Long Non-Coding RNA Transcriptomes
Source: PLoS One. 2011 Oct 3;6(10):e25915. doi: 10.1371/journal.pone.0025915 (PMC3185064; doi:10.1371/journal.pone.0025915)

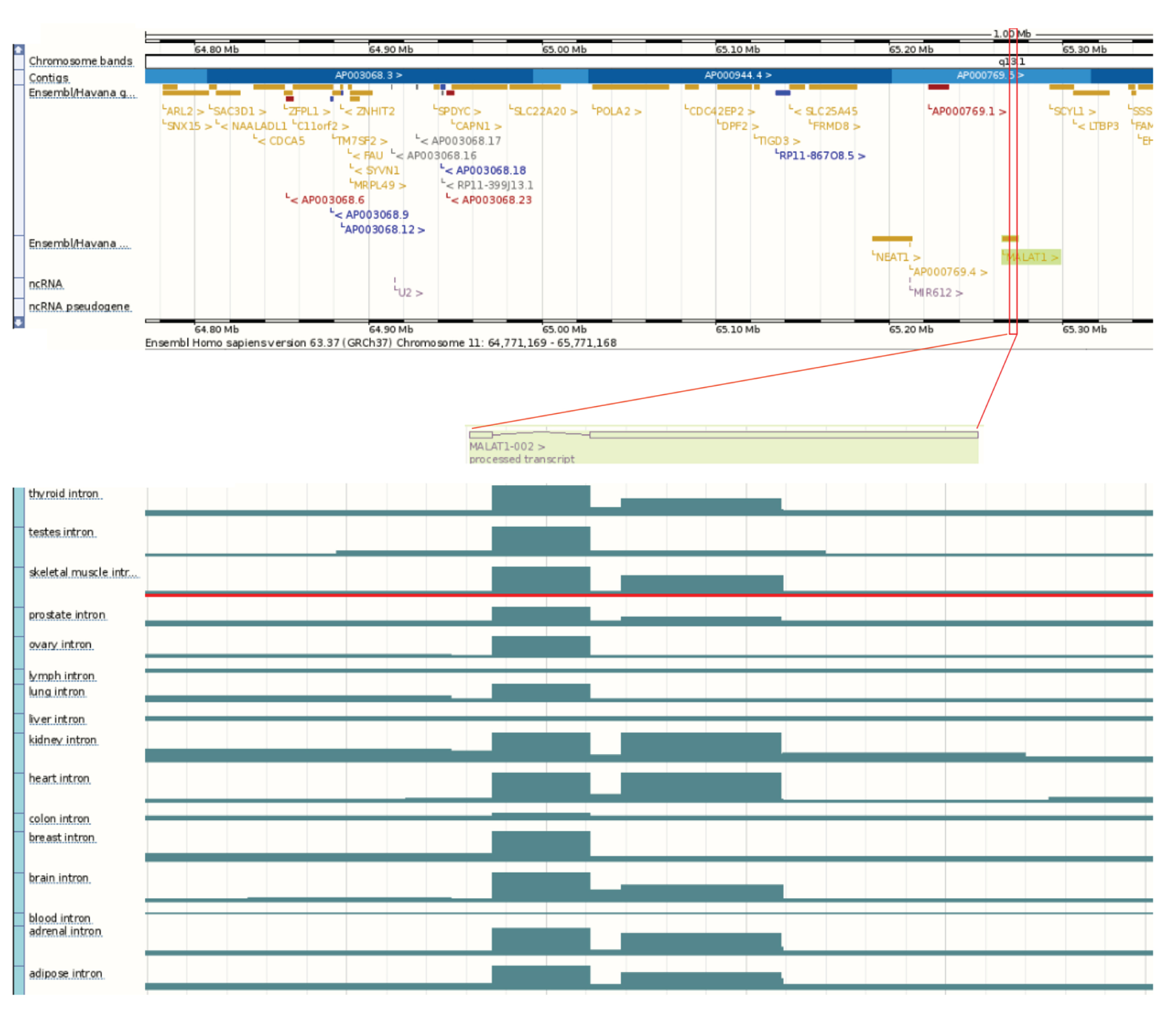

Supplement: Figure S1 — Tissue expression profiles of MALAT1. Expression was derived from the Human BodyMap 2.0 RNASeq track in Ensembl v62. (JPG) [file pone.0025915.s001.jpg]

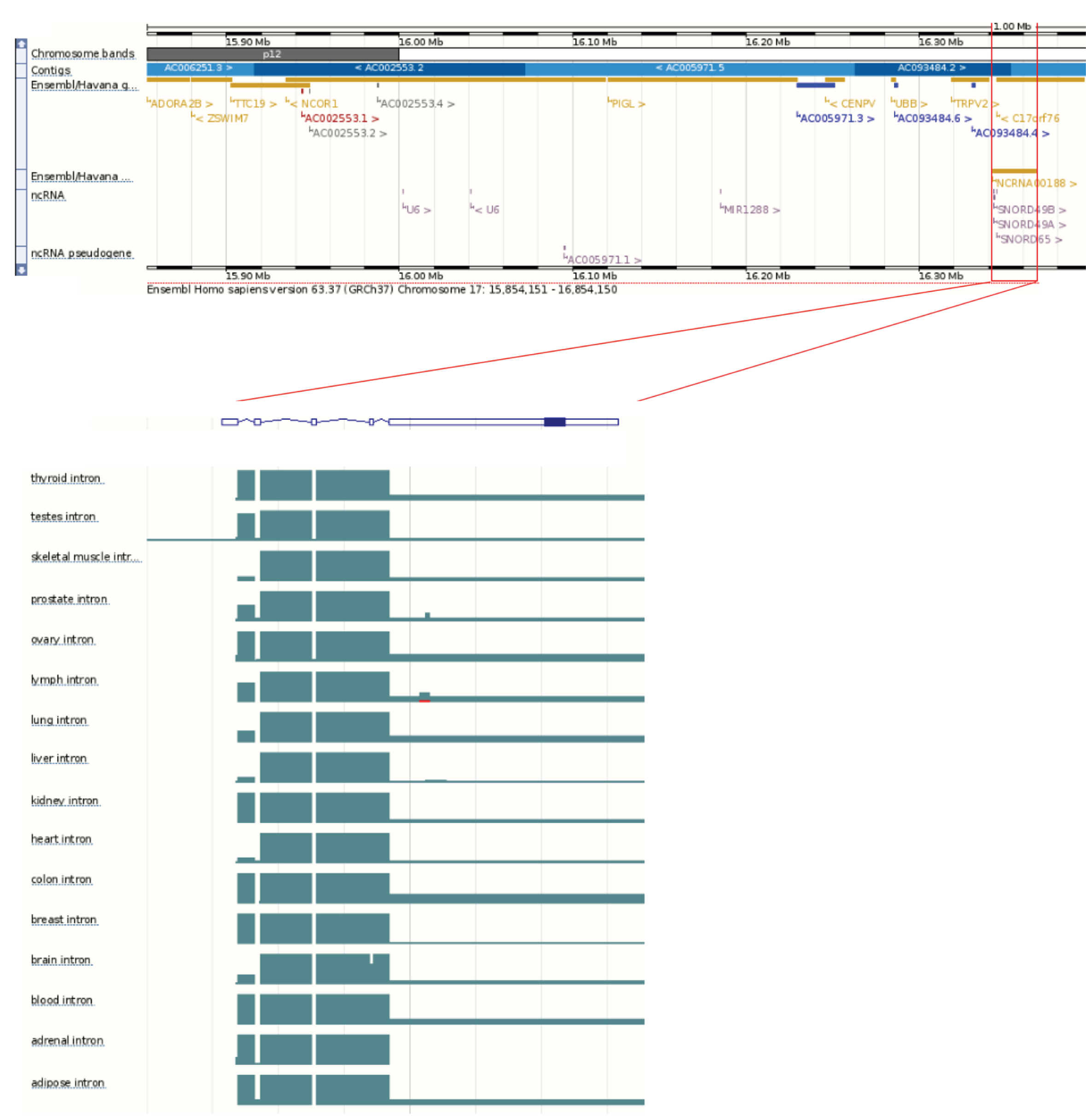

Supplement: Figure S2 — Tissue expression profiles of NCRNA00188. Expression was derived from the Human BodyMap 2.0 RNASeq track in Ensembl v62. (JPG) [file pone.0025915.s002.jpg]

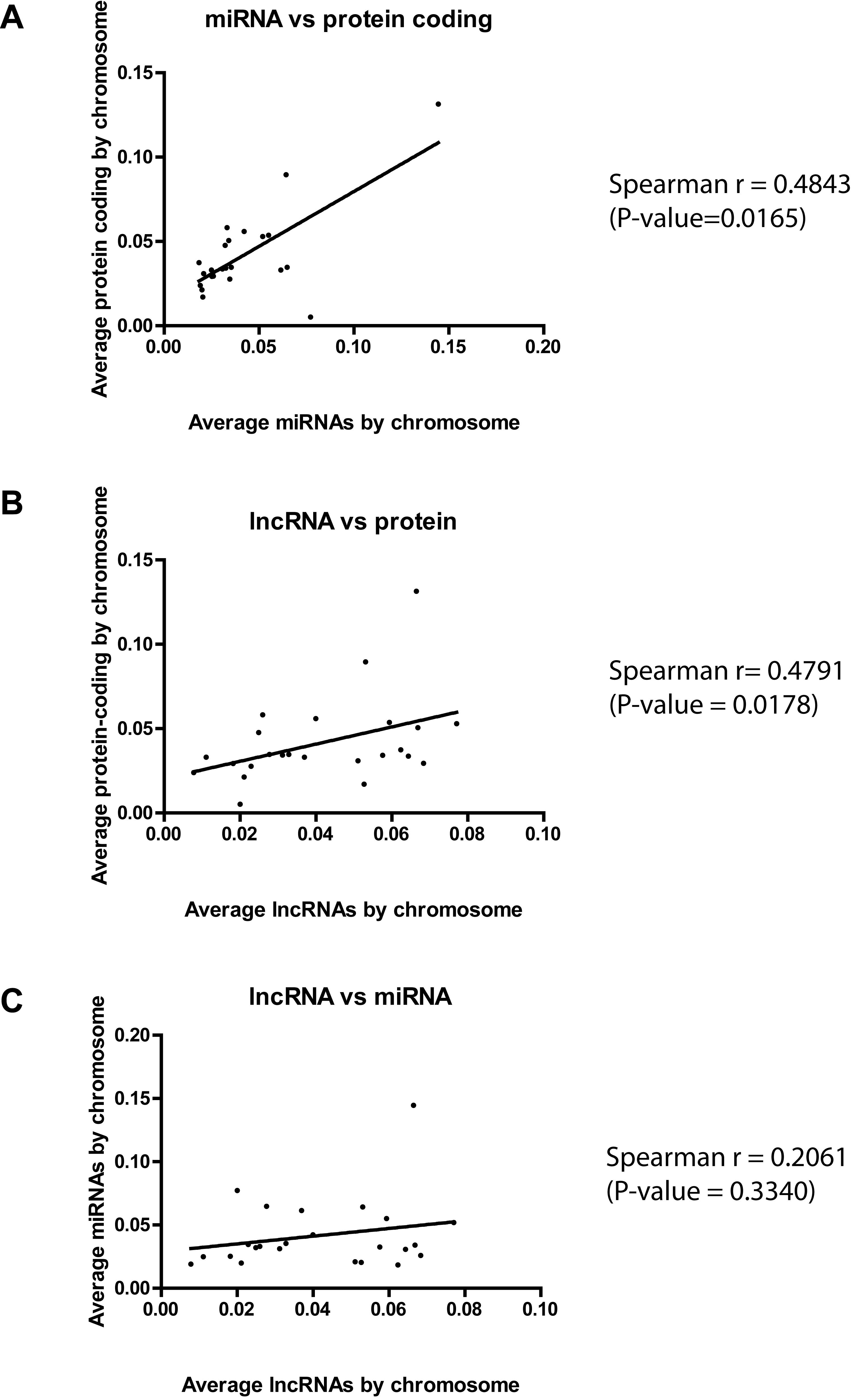

Supplement: Figure S3 — Correlation of chromosome distribution between protein-coding genes, miRNAs and lncRNAs. (A) Protein-coding genes compared to miRNAs, (B) Protein-coding genes compared to lncRNAs, (C) lncRNAs compared to miRNAs. The chromosome locations of protein-coding genes (n = 20,655), microRNAs (n = 1746) and long non-coding RNAs (n = 9,891) were downloaded from Ensembl v62. The graphs were generated using GraphPad Prism. (JPG) [file pone.0025915.s003.jpg]
