# Supplementary material for: Human Cancer Long Non-Coding RNA Transcriptomes
Source: PLoS One. 2011 Oct 3;6(10):e25915. doi: 10.1371/journal.pone.0025915 (PMC3185064; doi:10.1371/journal.pone.0025915)
Supplement: Table S3 — SAGE library information. (DOC) [file pone.0025915.s006.doc]

Supplementary Table 3 - SAGE library information

| **Tissue or cell line** | **Total raw tag count** | **Percent total tags (%)** | **Cancer libraries** | **Percent cancer tags** | **Cell line libraries** | **Percent cell line tags** |
| --- | --- | --- | --- | --- | --- | --- |
| Bone Marrow | 61519 | 0.3 | 1 | 100 | 0 | 0 |
| Brain | 6701431 | 27.3 | 71 | 91.2 | 15 | 18.4 |
| Breast | 3912333 | 15.9 | 35 | 56.8 | 4 | 6.0 |
| Cartilage | 707207 | 2.9 | 8 | 100 | 7 | 100 |
| Colon | 602215 | 2.5 | 9 | 100 | 8 | 100 |
| Embryonic Stem Cells | 2594061 | 10.6 | 0 | 0 | 11 | 100 |
| Esophagus | 302942 | 1.2 | 3 | 75.0 | 0 | 0 |
| Retina | 994714 | 4.1 | 1 | 8.8 | 0 | 0 |
| Gall Bladder | 312622 | 1.3 | 3 | 72.6 | 0 | 0 |
| Heart | 83063 | 0.3 | 0 | 0 | 0 | 0 |
| Kidney | 100281 | 0.4 | 1 | 100 | 0 | 0 |
| Liver | 234442 | 1.0 | 3 | 71.7 | 2 | 46.0 |
| Lung | 4938630 | 20.1 | 13 | 40.2 | 0 | 0 |
| Lymph Node | 171434 | 0.7 | 1 | 58.0 | 0 | 0 |
| Muscle | 169106 | 0.7 | 1 | 36.2 | 0 | 0 |
| Ovary | 184755 | 0.8 | 3 | 100 | 2 | 71.0 |
| Pelvis | 83577 | 0.3 | 1 | 100 | 0 | 0 |
| Placenta | 309025 | 1.3 | 0 | 0 | 0 | 0 |
| Peritoneum | 53527 | 0.2 | 0 | 0 | 0 | 0 |
| Prostate | 520344 | 2.1 | 4 | 48.7 | 4 | 51.1 |
| Reference RNA (CL) | 51729 | 0.2 | 0 | 0 | 1 | 100 |
| Spinal Cord | 54785 | 0.2 | 0 | 0 | 0 | 0 |
| Stomach | 475267 | 1.9 | 6 | 84.6 | 0 | 0 |
| Testis | 66352 | 0.3 | 1 | 100 | 0 | 0 |
| Thyroid | 342180 | 1.4 | 2 | 51.8 | 0 | 0 |
| Vascular | 384734 | 1.6 | 0 | 0 | 0 | 0 |
| White Blood Cells | 832586 | 3.4 | 0 | 0 | 0 | 0 |
| Total | 24436076 | 100% | 167 | n/a | 54 | n/a |

CL = Cell line
